# Mechanisms of Cancer Cell Killing by the Adenovirus E4orf4 Protein

**DOI:** 10.3390/v7052334

**Published:** 2015-05-07

**Authors:** Tamar Kleinberger

**Affiliations:** Department of Microbiology, Faculty of Medicine, Technion—Israel Institute of Technology, 1 Efron St., Bat Galim, Haifa 31096, Israel; E-Mail: tamark@tx.technion.ac.il; Tel.: +972-48-295-257

**Keywords:** adenovirus, E4orf4, protein phosphatase 2A (PP2A), Src kinase, actin, Ynd1, Golgi UDPase, Acf1, SNF2h, cell-death, cell cycle, chromatin remodeling

## Abstract

During adenovirus (Ad) replication the Ad E4orf4 protein regulates progression from the early to the late phase of infection. However, when E4orf4 is expressed alone outside the context of the virus it induces a non-canonical mode of programmed cell death, which feeds into known cell death pathways such as apoptosis or necrosis, depending on the cell line tested. E4orf4-induced cell death has many interesting and unique features including a higher susceptibility of cancer cells to E4orf4-induced cell killing compared with normal cells, caspase-independence, a high degree of evolutionary conservation of the signaling pathways, a link to perturbations of the cell cycle, and involvement of two distinct cell death programs, in the nucleus and in the cytoplasm. Several E4orf4-interacting proteins including its major partners, protein phosphatase 2A (PP2A) and Src family kinases, contribute to induction of cell death. The various features of E4orf4-induced cell killing as well as studies to decipher the underlying mechanisms are described here. Many explanations for the cancer specificity of E4orf4-induced cell death have been proposed, but a full understanding of the reasons for the different susceptibility of cancer and normal cells to killing by E4orf4 will require a more detailed analysis of the complex E4orf4 signaling network. An improved understanding of the mechanisms involved in this unique mode of programmed cell death may aid in design of novel E4orf4-based cancer therapeutics.

## 1. Introduction

The adenovirus (Ad) Early region 4 open-reading-frame 4 (E4orf4) protein is a regulator of the progression of Ad infection from the early to the late phase. Its activities include down-regulation of early viral gene expression and of cellular genes affecting Ad replication [1,2,3,4], control of alternative splicing of viral mRNAs [5,6], and regulation of protein translation [4,7]. E4orf4 effects on viral gene expression also influence viral DNA replication [8,9]. Deletion of the E4orf4 sequence from the Ad genome was shown to moderately inhibit Ad replication in cells in tissue culture by only a few fold [7,10]. However, considerable homology exists between E4orf4 proteins belonging to several classes of human Ads [11], suggesting that E4orf4 function is important for the virus. The effect of E4orf4 on the efficiency of Ad infection in a whole organism is currently unknown.

**Figure 1:**
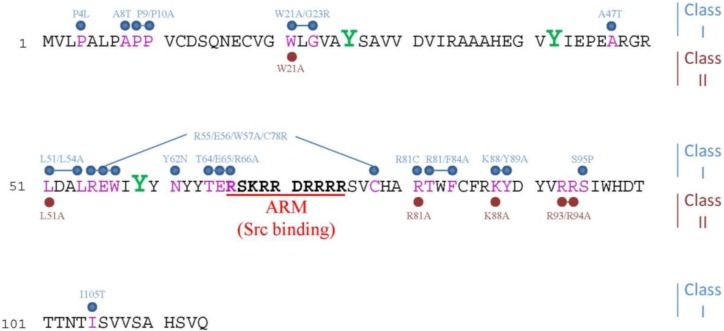
Mutation analysis of PP2A and Src binding sites in E4orf4. The Ad5 E4orf4 protein sequence is shown. The basic E4ARM domain is displayed in bold and underlined in red. The tyrosines that are phosphorylated by Src kinases are in bold green and a larger font. Other residues involved in E4orf4-induced cell death are marked in pink. Mutations in the E4orf4 sequence that reduced association of E4orf4 with a PP2A phosphatase activity by at least two-fold and impaired E4orf4-induced cell death (‘class I’ mutants [11]) are shown above the sequence in light blue. Mutations that did not reduce the E4orf4-PP2A interaction more than two-fold but were deficient in induction of cell death (‘class II mutants’) are shown below the sequence in dark red. Three more mutations were found to reduce PP2A binding: V19A/T102I, A25T/ΔD52/R87C and ΔV29/R81C, which are not shown for simplicity sake. The basic E4orf4 ARM domain is required for Src kinase binding, but has only a low effect on PP2A binding [14,19].

The E4orf4 protein is a 14 kDa polypeptide without known non-Ad homologs. *Ab initio* modeling of the structure of E4orf4 predicted that it consists of three α-helices, as well as N- and C-terminal loops [12]. The E4orf4 protein contains a highly basic stretch of amino acids (residues 66–75), which may provide a nuclear and nucleolar targeting function [13], as well as a docking site for one of the E4orf4 partners, Src kinase [14] (Figure 1).

Protein phosphatase 2A is a major E4orf4 partner [15]. Phosphatases of the PP2A group are Ser/Thr phosphatases, which are involved in most cellular processes. These enzymes contain three subunits: one of two isoforms of a catalytic C subunit encoded by *PPP2CA* and *PPP2CB*, one of two isoforms of a scaffolding A subunit encoded by *PPP2R1A* and *PPP2R1B*, and one of twenty-three regulatory B subunits belonging to four unrelated gene families, each containing several isoforms (B/B55: *PPP2R2A-PPP2R2D*, B’/B56: *PPP2R5A-PPP2R5E*, B’’: *PPP2R3A-PPP2R3C*, and B’’’: *STRN*, *STRN3*, *STRN4*). The combinatorial assembly of the various PP2A subunits yields 92 combinations of heterotrimeric enzyme complexes (ABC) and four combinations of heterodimeric complexes (AC), each possessing its own catalytic properties, substrate specificities, tissue or cell-specific expression, and subcellular localization [16]. A functional heterodimeric BC complex has also been described [17]. It has been reported that in both mammalian cells and in yeast E4orf4 associates with PP2A through an interaction with regulatory B subunits, including the mammalian B55/yeast Cdc55 and mammalian B56 subunits [11,15,18,19,20,21]. The E4orf4-PP2A complex contains all three types of PP2A subunits (ABC) and has substantial phosphatase activity [15,18,21]. All E4orf4 activities during virus infection that are known to date require an interaction between PP2A and the viral protein [2,3,5,7,11,15].

## 2. The Unique Mode of E4orf4-Induced Cell Death

When E4orf4 is expressed alone, outside the context of virus infection, it induces cell death in transformed cells, which is dose-dependent [20,22], p53-independent [23,24,25] and possesses several interesting features described below. These include a higher susceptibility of cancer cells to E4orf4-induced cell killing compared with normal cells, participation of caspase-independent signaling pathways that are linked downstream to known cell death programs, a high degree of evolutionary conservation of the relevant signaling pathways, a link to perturbations of the cell cycle, and the involvement of at least two distinct cell death programs, in the nucleus and in the cytoplasm. In contrast, during virus infection E4orf4 does not contribute significantly to cell death for several reasons: (1) E4orf4 levels during virus infection are regulated by negative feedback [2] and may not be sufficient for induction of cell death; (2) Viral cell death-inhibitory genes may reduce premature cell killing by E4orf4; (3) During infection, E4orf4 is detected mostly in the nucleus [26], suggesting that at least the cytoplasmic cell death program induced by E4orf4 may be diminished; (4) In nature Ad infects normal, untransformed cells that may be less susceptible to E4orf4 toxicity. Moreover, E4orf4 was reported to possess a protective effect during Ad infection of non-transformed CREF cells [4].

Two major E4orf4 partners, protein phosphatase 2A (PP2A) and Src kinases, together with additional E4orf4-associating proteins, play an important role in E4orf4-induced cell death, and studies of their contribution are described in detail below (Section 3).

### 2.1. Cancer Specificity of E4orf4-Induced Cell Death

Experiments in primary rat embryo fibroblasts revealed that E4orf4 induced high levels of cell death when co-transfected with various oncogene combinations but not when co-transfected with an empty vector. Thus oncogene expression sensitized primary cells grown in tissue culture to killing by E4orf4 [19]. A mutant E4orf4 protein that lost the ability to bind an active PP2A also lost the ability to kill oncogene-transformed cells [19]. Consistent with these results, a later review paper described studies showing that E4orf4 induced cell death in 40 human cancer cell lines, but not in several primary human cell types derived from various tissues [27]. Unpublished results from our laboratory indicate that E4orf4 also eliminates cancer clones in the *Drosophila* eye disc more efficiently than normal clones, suggesting that E4orf4-induced cell death is cancer specific not only in tissue culture cells but also in a multicellular organism.

The basis for the differential response of normal and cancer cells to E4orf4 is not clear yet but several possible explanations have been proposed based on the nature of transformed cells and on features of E4orf4-induced cell death described below: (1) Activation of the oncogenic state leads to induction of latent apoptotic signals that are uncoupled from the basic apoptotic machinery and provide a lower threshold for activation of cell death by various signals [28]; (2) It was reported that cancer cells become addicted to crucial oncogenic pathways [29] and it may be possible that E4orf4 inhibits these pathways leading to cell death of the oncogene-addicted cells but not of normal cells; (3) E4orf4 may exploit activated oncogenes in cancer cells, such as Src, for induction of cell death (Section 3.5); (4) Cell cycle checkpoints in cancer cells are defective to some extent [30] and these cells would be more susceptible to E4orf4, which disrupts mitotic checkpoints (Section 2.5); (5) We showed in the *Drosophila* model system that E4orf4 can inhibit classical apoptosis in normal fly tissues (Section 4), and it can be hypothesized that this E4orf4 function is lost in cancer cells, leading to a more effective cell killing [31]; (6) E4orf4-induced structural changes observed in mitochondria (Section 3.5) could affect metabolic reprogramming, which may influence cancer and normal cells differentially [32]. Deciphering the mechanisms underlying E4orf4-induced cell death will facilitate a better understanding of the different susceptibility of normal and cancer cells to E4orf4 toxicity.

As E4orf4 induces a p53-independent, non-canonical programmed cell death [23,24,25] and a large percent of human tumors are p53-deficient [33], investigation of the unique mode of E4orf4-induced cancer cell killing may have exciting implications for cancer therapy. Although understanding of the differential sensitivity of normal and cancer cells to E4orf4-induced cell death is still minimal, several researchers began to explore the feasibility of using E4orf4-based approaches for cancer therapy [34,35,36,37,38]. These initial attempts to use E4orf4 to treat cancer cells *in vivo* are still very preliminary but they provide further motivation to develop basic research aimed at understanding the E4orf4 cell death network both in tissue culture cells and in animal models.

### 2.2. Caspase-Independent Cell Death Signaling that Feeds into Known Cell Death Pathways

Several reports have indicated that E4orf4-induced cell death is caspase-independent although crosstalk with caspase-dependent pathways also occurs [23,39,40,41]. Numerous lines of evidence led to these conclusions. First, addition of various broad-range caspase inhibitors did not prevent E4orf4-induced cell killing in several cell lines and in some cases no caspase activation was observed upon E4orf4 expression [23,41]. However, caspase activation was observed when E4orf4 was expressed in other types of cells [40,41]. Furthermore, in some cell lines caspase inhibition eliminated certain morphologies associated with E4orf4-induced cell death, such as accumulation of sub-G1 cells containing fragmented DNA [40,41], and in other cases caspase inhibition decreased nuclear condensation induced by E4orf4 and even increased cell survival measured by a clonogenic assay [40]. Variability in the type of caspase-dependent pathways that were induced by E4orf4 also emerged from these studies. One report described a contribution of the extrinsic apoptotic pathway including FADD/MORT1 and caspase-8 to E4orf4-induced cell death in 293T cells, but no involvement of caspase 9 [40], whereas another report described a contribution of the mitochondria-apoptosome intrinsic apoptotic pathway to E4orf4-induced DNA fragmentation in C33A cells [41].

The activation of classical caspase-dependent pathways in addition to caspase-independent cell killing may facilitate amplification of E4orf4-induced cell death. Furthermore, in addition to utilizing classical apoptosis to assist E4orf4-induced cell killing in some cell lines, it was reported that in the p53-deficient H1299 cells E4orf4 could execute cell death with necrotic characteristics through induction of mitotic catastrophe [42]. Taken together, the results suggest that E4orf4 initiates caspase-independent signaling that can be linked to various cell death pathways, such as caspase-dependent apoptosis or mitotic catastrophe-mediated necrosis. A crosstalk between caspase-dependent and independent pathways was also observed during E4orf4-induced cell death in *Drosophila melanogaster*, and both these types of cell death contributed to E4orf4-induced toxicity in the fly [22]. The mechanisms underlying the decision dictating the direction in which caspase-independent E4orf4 cell death signaling will proceed are not known, but they may depend on the physiological state of the cells and on the cell genetic or proteomic content.

### 2.3. Evolutionary Conservation of E4orf4 Cell Death Signaling

**Figure 2:**
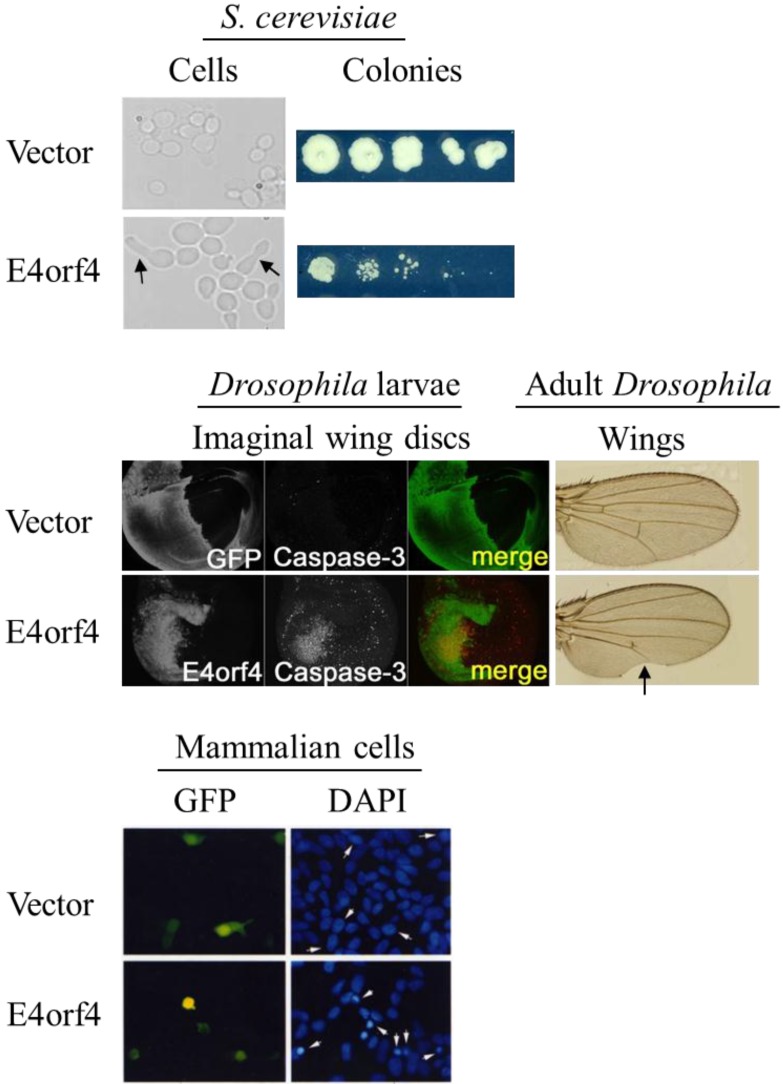
E4orf4-induced cell death is highly conserved in evolution. E4orf4-induced toxicity was studied in the yeast *S. cerevisiae*, in *Drosophila melanogaster* and in mammalian cells in tissue culture. The pictures shown here demonstrate E4orf4 effects in the various organisms, including budding defects in yeast (marked with arrows), reduced size of yeast colonies, caspase activation in a *Drosophila* wing disc, a wing defect in adult flies (marked with an arrow) and nuclear condensation seen by DAPI staining with cell rounding and blebbing seen by GFP staining in mammalian cells (arrows mark nuclei of transfected cells). E4orf4-induced toxicity required an interaction with PP2A in all these organisms and an interaction with Src kinases contributed to this process in flies and mammals. Studies of E4orf4 in all these model systems contributed different insights into E4orf4 biology. See the text for more details. Parts of this figure were adapted from the following sources with permissions: Top panel: [45]. Middle panel: [22]. Bottom panel: [25].

The major E4orf4 partners described to date are protein phosphatase 2A (PP2A) and Src kinases, and their contribution to E4orf4-induced cell death signaling is described below (Section 3). PP2A is highly conserved in evolution from yeast to mammalian cells [43], and Src kinase, although absent from yeast, is conserved from the unicellular choanoflagellate *Monosiga ovata* through the primitive multicellular sponge *Ephydatia fluviatilis* to mammals [44]. It appears that the high evolutionary conservation of the E4orf4 partners results in the ability of E4orf4 to induce its unique mode of cell death signaling in various organisms from yeast [18,45] through *Drosophila* [22] to mammalian cells (Figure 2), facilitating the use of model organisms for research on E4orf4 functions.

### 2.4. Morphological Hallmarks of E4orf4-Induced Cell Death and Assays for Measuring E4orf4-Induced Cell Killing

Since upstream caspase-independent events in the E4orf4-induced cell death pathway appear to be linked downstream to various types of cell death, only few morphological hallmarks of E4orf4-induced cell killing can be consistently used to assay this process. It has been shown that the most typical morphologies associated with E4orf4-induced cell death include membrane blebbing, nuclear condensation and cell detachment, whereas morphologies associated with classical apoptosis such as DNA fragmentation, caspase activation, phosphatidylserine externalization, or changes in mitochondrial membrane potential do not always accompany E4orf4-induced cell killing [23,40,42]. Therefore, the assays commonly used to quantify E4orf4-induced cell death include determination of the percentage of cells with membrane blebbing or with nuclear condensation and clonogenic cell survival assays.

### 2.5. Perturbations of the Cell Cycle Precede E4orf4-Induced Cell Death

Many adenoviral genes cause alterations in the cell cycle as a consequence of their contribution to virus infection [46]. When cellular effects of E4orf4 were examined, it was noticed that induction of E4orf4 expression in HEK293-derived cell lines resulted in accumulation of cells with 4N DNA content 24 h after induction, suggesting a cell cycle arrest at G2/M. This arrest was followed at later times by an accumulation of apoptotic sub-G1 cells [25,45]. Later studies of E4orf4 in the yeast *S. cerevisiae* revealed that E4orf4 induced an irreversible growth arrest in this organism, which was also associated with G2/M arrest [18,45]. Visualization of spindles with GFP-marked tubulin demonstrated that most E4orf4-expressing yeast cells were arrested either with short pre-anaphase spindles or with extended telophase spindles [45]. At least part of the inhibitory effect of E4orf4 on cell cycle progression in yeast could be attributed to its interaction with the spindle checkpoint containing the anaphase-promoting complex/cyclosome (APC/C) and recruitment of PP2A to this complex [45]. APC/C is an E3 ubiquitin ligase, which marks cell cycle regulators for degradation. It associates with various activating subunits at specific stages of the cell cycle, including Cdc20 early in mitosis and Cdh1 in later mitosis and G1 [47]. Ase1 and Pds1 are substrates of APC/C^Cdh1^ and APC/C^Cdc20^ respectively. It was shown that E4orf4 stabilized Ase1 and increased Pds1 levels [45], suggesting that it inhibited both APC/C complexes. Furthermore, benomyl, a microtubule depolymerizing drug that transiently triggers the spindle assembly checkpoint by inhibiting APC/C^Cdc20^ sensitized yeast cells to E4orf4 toxicity [45]. This observation is consistent with inhibition of APC/C^Cdc20^ by E4orf4, as benomyl may exacerbate cell cycle arrest when APC/C is already partially inhibited by E4orf4. Moreover, using the power of yeast genetics it was shown that mutations in APC/C and its co-activators Cdc20 and Cdh1 displayed synthetic lethality with E4orf4 expression [45], further demonstrating that APC/C inhibition increased E4orf4 toxicity.

Additional cell cycle mutants that manifested synthetic lethality with E4orf4 expression in yeast contained mutations that reduced activity of Cdc28, the yeast cell cycle-dependent kinase 1 (Cdk1) [45]. Furthermore, E4orf4 increased Histone H1 phosphorylation by a Cdc28 complex containing the mitotic Clb2 cyclin and this effect was dependent on the yeast B55 subunit of PP2A, Cdc55, and on the Cdc28 phosphatase Mih-1 [18,45]. It was suggested that E4orf4 may partially counteract its own cell cycle inhibitory effect by stimulating Cdc28, an activator of APC/C [48,49], as part of a multifaceted mode of regulation [45].

In contrast to the results indicating inhibition of APC/C^Cdc20^, it was reported that when yeast cells were arrested in S-phase by hydroxyurea treatment, APC/C^Cdc20^ was prematurely activated by E4orf4 and Pds1 levels were reduced, as were the levels of Scc1 whose stability depends on the presence of Pds1. The activity of APC/C^Cdh1^ was not altered under these conditions. Premature activation of APC/C^Cdc20^ required the E4orf4 interaction with PP2A [50]. How may these seemingly contradictory results describing both inhibition and activation of APC/C^Cdc20^ be reconciled? A possible explanation could involve the existence of different potential targets of PP2A in the APC/C complex and in its modulators that may be targeted differentially by the E4orf4-PP2A complex in S-phase and mitosis. For example, the APC/C inhibitor Emi1, which is itself inhibited by Cdk1 phosphorylation [51] could be such a target. Although Emi1 is not normally phosphorylated during S-phase, a Cdc55-dependent E4orf4-induced Cdk1 activation [18,45,50] may lead to Emi1 phosphorylation in S-phase followed by premature APC/C activation. On the other hand, during mitosis, Cdc55-dependent inhibition of APC/C may be more prominent [45], while Emi1 is degraded at mitotic entry [52,53]. However, whereas inhibition of APC/C complexes may account at least in part for the E4orf4-induced G2/M arrest, it is not clear how premature activation of APC/C^Cdc20^ in S-phase assists in this process. Thus a better understanding of the interaction between E4orf4 and the cell cycle requires more study.

The delay in the cell cycle and M-phase perturbations caused by E4orf4 may result in cells with improper chromosome numbers returning to the cell cycle, leading to cell death. It was indeed shown recently that E4orf4 expression in H1299 cells led to accumulation of cells with 4N DNA content which contained high levels of Cyclin E but not of mitotic markers such as Cyclin B1 or pSer10 Histone H3. It was suggested that these cells were in the G1 phase of the cell cycle rather than in G2/M and that they progressed from mitosis to G1 in the absence of cytokinesis [54]. These results were further confirmed by time-lapse microscopy experiments demonstrating that E4orf4 slowed down dramatically the transit of H1299 cells through mitosis and caused a delay or failure of cytokinesis [55]. E4orf4-induced perturbation of cytokinesis was also observed in yeast cells, which displayed abnormal budding [17,18,45] (Figure 2). Both the tetraploid H1299 cells and cells with 2N DNA content found in the E4orf4-expressing cell population proceeded to undergo cell death, as measured by uptake of propidium iodide. When arrested at G0/G1 and then released into the cell cycle, E4orf4-expressing cells also manifested a deficiency in initiation of DNA replication [54]. DNA replication may be affected by E4orf4 through inhibition of APC/C^Cdh1^, as this complex is involved in degradation of proteins that control DNA replication, or through targeting of PP2A to other substrates involved in regulation of DNA replication [56,57].

The findings portrayed above, demonstrating that the activity of Cdc28 in complex with mitotic cyclins was increased in E4orf4-expressing yeast cells whereas E4orf4-expressing H1299 cells contained high levels of Cyclin E but not of the mitotic Cyclin B1 [18,45,54] indicate that these events may depend on the type of cell expressing E4orf4. However, taken together, the results indicate that E4orf4 targets various cell cycle regulators and that perturbation of the normal cell cycle may lead to cell death. A link between cell cycle disruption and induction of cell death was shown previously, using drugs or other viral genes that induced both cell cycle arrest and cell death (for example, [58,59,60,61]), but the exact mechanisms underlying this link have not been fully defined yet. Mitotic catastrophe was suggested as one mechanism that can account for the link between cell cycle perturbations and cell death [62,63]. Furthermore, mitotic catastrophe can lead to various modes of cell death, such as apoptosis and necrosis, as well as to senescence, depending on the genetic identity of the cells and the physiological conditions [64]. Besides mitotic catastrophe, other distinct mechanisms can remove cells that failed at later mitotic stages and progressed to G1, such as outcompeting cells with an abnormal number of chromosomes, a condition that leads to reduced fitness [65]. Since E4orf4 interacts with other nuclear proteins which contribute to induction of cell death, besides direct cell cycle regulators [57], and because it also induces a cytoplasmic cell death program (Section 2.6), additional cell death pathways are also likely to be important for E4orf4 toxicity.

### 2.6. E4orf4 Induces Nuclear and Cytoplasmic Cell Death Programs

During virus infection, GFP-tagged E4orf4 was described to concentrate in the nucleus but its localization did not overlap viral replication centers. Only very little E4orf4 staining was found in the cytoplasm [26]. The localization of native E4orf4 during virus replication was not published. When E4orf4 expression was induced outside the context of virus infection, several studies in various cell lines revealed that it first accumulated in the nucleus but was later detected in the cytoplasm, cytoskeleton and membrane regions, with a tendency to concentrate in membrane blebs [1,13,41,66]. Nuclear localization of E4orf4 required a highly basic arginine-rich motif (residues 66–75) named E4ARM [13] (Figure 1). This motif was reported to target GFP-tagged E4orf4 to nucleoli as well [13], although native E4orf4 was not observed to concentrate there [67].

When expressed alone, the shift of E4orf4 accumulation from nuclear to non-nuclear cellular locations was shown to depend on the interaction of E4orf4 with Src family kinases and on Tyr-phosphorylation of E4orf4 by these kinases [66,68]. Co-expression with constitutively active Src kinase increased E4orf4 accumulation in the cytoplasm, in membrane blebs and in a triton-insoluble cytoskeleton protein fraction [66]. Substitution of E4orf4 Tyr residues, which are normally phosphorylated by Src kinases, to phenylalanine led to E4orf4 nuclear localization, whereas mimicking phosphorylation by replacing Tyr residues with glutamic acid enhanced E4orf4 accumulation outside the nucleus [68]. Based on these results, it was suggested that E4orf4 phosphorylation by Src may result in changes to E4orf4 conformation in such a way that would promote its retention outside the nucleus. It was reported that leptomycin B, an inhibitor of CRM1-mediated export from the nucleus did not affect E4orf4 cytoplasmic accumulation, but inhibition of the myosin II motor by blebbistatin induced E4orf4 nuclear retention [41]. These results imply that the myosin II motor, but not CRM1-dependent nuclear export, is required for the accumulation of E4orf4 outside the nucleus.

Because E4orf4 shifted with time from a nuclear to a cytoplasmic-membranal localization, experiments were performed to determine the relative contribution of nuclear, cytoplasmic, and membranal E4orf4 to cell death signaling [41]. Targeting GFP-tagged E4orf4 to the cytoplasm (using a nuclear export sequence) or to membranes (using a myristoylation signal or a CAAX box) reconstructed efficiently the contribution of Src to E4orf4-induced cell death, inducing membrane blebbing and nuclear condensation at least as competently as the WT GFP-E4orf4 protein. Nuclear targeting of GFP-E4orf4 (using a nuclear localization sequence) prevented the early appearance of membrane blebbing and delayed the appearance of nuclear condensation. It was further shown, using E4orf4 Tyr substitutions, that E4orf4 phosphorylation by Src was required for the cytoplasmic morphologies associated with E4orf4-induced cell death, such as membrane blebbing, but had a much smaller effect on nuclear condensation [68]. Analysis of additional E4orf4 mutants revealed that E4orf4 binding to its major partners, PP2A and Src kinases, affected differentially the nuclear and cytoplasmic forms of E4orf4-induced cell death [14]. Based on these results it was suggested that E4orf4 induced two distinct cell death programs, a nuclear program supported by the interaction with PP2A and a cytoplasmic program supported by the interaction with Src kinases. Caspase activation occurred, but was dispensable for the execution of both cell death programs, whereas calpains were required for the Src-mediated cytoplasmic death signal. It was further shown that the relative contribution of the two branches of E4orf4-induced cell death differed in various cell lines [41]. However, it is still not absolutely clear whether E4orf4 activates two independent cell death programs or whether initial E4orf4 functions in the nucleus contribute to activation of both pathways. Thus, artificial targeting of E4orf4 to various cellular sites was not absolute and the residual presence of E4orf4 in the nucleus may have been sufficient to perform the required preliminary nuclear functions that may potentially be needed to trigger both branches of the death program. Likewise, although mutants that did not bind PP2A were still able to induce cytoplasmic cell death signaling [14], it is possible that residual PP2A binding was sufficient to initiate it. Although E4orf4-induced cell death was shown to be dose-dependent [20,22], it was not determined yet whether both PP2A- and Src-dependent death programs required high E4orf4 levels or whether even low levels of interaction with PP2A were enough to initiate cell death signaling.

## 3. Mechanisms Underlying the Contribution of E4orf4-Associating Proteins to E4orf4-Induced Cell Death

### 3.1. PP2A

Early studies of E4orf4-induced cell death addressed the question whether the interaction with PP2A was required for this process. Mutation analyses of E4orf4 (Figure 1) revealed that alterations in several residues significantly reduced PP2A binding and also extensively decreased E4orf4 toxicity [11,19]. These mutants were classified as class I mutants. On the other hand, a second class of E4orf4 mutants, class II, was also described, which contained mutants that bound PP2A at more than 50% WT efficiency but their ability to induce cell death was significantly impaired [11]. It was suggested that the E4orf4-PP2A interaction of class II mutants was different than the WT interaction and could not exert a biological function. It is also possible that these mutants failed to bind E4orf4-PP2A substrates or that they failed to bind another protein that might also contribute to induction of cell death. A more direct support for a role of the PP2A-B55 subunit, which mediates the PP2A-E4orf4 interaction, in E4orf4-induced cell death came from the findings that a B55 antisense construct that reduced B55 expression also inhibited E4orf4- but not p53-induced cell death [19] and that B55 overexpression increased E4orf4 toxicity [12,21,45]. Similarly, in the yeast *S. cerevisiae*, deletion of *cdc55*, encoding the yeast PP2A B55 subunit, or of *tpd3*, encoding the PP2A-A subunit, reduced dramatically E4orf4 toxicity [18,45]. Although E4orf4 was reported to bind not just the B55 subunit of PP2A but also the B56 subunit [21], only PP2A complexes containing the B55 subunit contributed to induction of cell death [18,21,45]. It appears that the complete PP2A holoenzyme plays a role in E4orf4 toxicity as it was reported that an E4orf4 mutant (S95P) that bound the B55 subunit alone, possibly displacing the A and C subunits from the complex, acted as a dominant negative mutant, reducing cell death induced by WT E4orf4 [21]. On the other hand, based on work in yeast, it was suggested that although the PP2A-C subunit contributes to E4orf4-mediated toxicity, the Cdc55 subunit may provide an additional contribution to this process that is independent of stable complex formation with the PP2A-C subunit [69]. However, Cdc55 did not appear to mediate E4orf4 toxicity through a Tpd3-independent pathway [69]. The nature of the PP2A-C-subunit-independent role of the Cdc55 subunit is not clear, although it was suggested that Cdc55 may form a heterotrimeric phosphatase with Tpd3 and with another PP2A-like catalytic subunit, Sit4, a yeast PP6 orthologue. Remarkably, deletion of Sit4 in yeast and knockdown of PP6 in mammalian cells increased E4orf4-mediated cell death. However, a physical interaction between E4orf4 and Sit4 could not be detected [69].

It was previously shown that the interaction of E4orf4 with an active PP2A was required for various E4orf4 functions during virus infection including down-regulation of gene expression and regulation of alternative splicing and that the PP2A inhibitor okadaic acid inhibited these E4orf4 functions [6,15]. However, the nature of the molecular consequences of the E4orf4-PP2A interaction that lead to cell death is controversial at present and two possibilities have been suggested. One suggestion indicates that upon overexpression, E4orf4 sequesters PP2A complexes containing the B55 subunit and prevents dephosphorylation of substrates that are required for cell survival [70]. Another hypothesis proposes that E4orf4 targets PP2A to novel substrates thus disrupting normal cellular regulation, creating optimal conditions for virus replication but leading to cell death in the absence of virus functions that may prevent premature cell killing.

The first hypothesis was based on the following findings: (1) Some, but not all, non-physiological substrates of the E4orf4-PP2A complex that were tested have been dephosphorylated *in vitro* less efficiently by an E4orf4-PP2A complex immunoprecipitated with an E4orf4-specific antibody than by a PP2A complex immunoprecipitated with HA-B55-specific antibodies in the absence of E4orf4 [20]. However, as acknowledged by the authors, E4orf4 associates with many PP2A complexes containing different B55 or B56 isoforms [20,21] and therefore this indirect comparison could reflect altered substrate specificity of the various holoenzymes associating with E4orf4; (2) Although E4orf4 expression in the context of the virus was associated with hypophosphorylation of some viral and cellular proteins [4,6], when E4orf4 was expressed alone hyperphosphorylation of some PP2A candidate substrates was observed [7,20]. However, this effect was not shown to be direct and may occur through activation of kinases, such as mTOR [7]; (3) Treatment of E4orf4-expressing cells with the PP2A inhibitors okadaic acid or I_1_^PP2A^ enhanced E4orf4-induced cell death [20]. However, okadaic acid can inhibit other PP2A-like phosphatases at least as efficiently as PP2A itself [71], and the I_1_^PP2A^ inhibitor was shown to associate with PP1 and modify its substrate specificity [72], indicating that these inhibitors may have off-target effects that are not dependent on PP2A. Indeed, as described above, reduced expression of PP6, a PP2A-like phosphatase, was shown to increase E4orf4-induced cell death [69], and PP6 is inhibited by okadaic acid even more efficiently than PP2A [73]. These observations suggest that the increase in E4orf4 toxicity that was reported to be caused by okadaic acid treatment may stem from PP6 inhibition; (4) The E4orf4-binding site in the PP2A-B55 subunit was mapped by bioinformatics predictions and mutation analyses [12,70]. These studies revealed that residues located on two sides of a putative substrate binding groove described in the PP2A-B55 structure [74] contributed to E4orf4 binding. Consequently it was suggested that E4orf4 competes with PP2A-B55 substrates for binding to the substrate binding site and prevents their dephosphorylation [70]. In support of this conclusion it was demonstrated that the presence of E4orf4 led to inhibition of p107 binding to B55 and to its hyperphosphorylation. On the other hand, only part of the B55 residues reported to be important for dephosphorylation of the PP2A-B55 substrate, Tau, were shown to be necessary for E4orf4 binding. This suggests that the binding sites for E4orf4 and PP2A substrates only partially overlap. Furthermore, during virus infection E4orf4 was shown to recruit PP2A-B55 to new substrates, such as a subgroup of the SR splicing factors [5,70], indicating that while E4orf4 may prevent accessibility of some substrates to PP2A, it also recruits novel substrates to this enzyme. In addition, the hypothesis that upon overexpression E4orf4 sequesters PP2A holoenzymes containing B55 and prevents dephosphorylation of important substrates, thus, leading to cell death, appears to contradict additional findings reported previously. First, overexpression of B55 increased E4orf4-induced cell death [12,21], while the sequestration hypothesis would predict that B55 overexpression would reduce cell killing. Second, knockdown of mammalian B55 and deletion of yeast Cdc55 inhibited E4orf4-induced cell killing instead of increasing it [18,21,45]. Therefore, a second hypothesis was proposed to explain PP2A-dependent E4orf4-induced cell death. This hypothesis postulates that E4orf4 recruits PP2A to novel substrates or enhances the affinity of substrate binding to PP2A, leading to an imbalance in normal cellular pathways and to cell death. In support of this hypothesis, overexpressed E4orf4, outside the context of virus infection, was reported to recruit PP2A to substrates such as the ACF chromatin remodeling factor in mammalian cells ([57], Section 3.2) and the mitotic regulator APC/C in yeast ([45], Section 3.3), both of which contributed to E4orf4 toxicity. Nevertheless, the possibility of a combined scenario may also be considered, in which the dephosphorylation of diverse proteins by PP2A is influenced in various ways by E4orf4 and the integrated effects of altered phosphorylation of these proteins lead to cell death. To further examine the proposed mechanisms underlying PP2A-dependent E4orf4 toxicity, direct E4orf4-PP2A targets must be identified and their contribution to the death process will have to be determined.

### 3.2. The ATP-Dependent Chromatin Remodeling Factor, ACF

**Figure 3:**
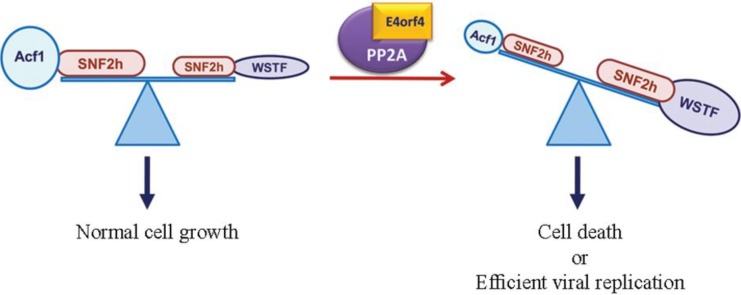
A working model of E4orf4 function in chromatin. Various SNF2h-containing complexes participate in chromatin remodeling and affect transcription, DNA replication, DNA repair, *etc.* The model shown in this figure proposes that E4orf4 together with PP2A inhibits the ACF chromatin remodeler. This inhibition leads to a shift in the balance between various SNF2h-containing remodeling complexes, allowing, for example, more activity of a WSTF-SNF2h complex. The variation in chromatin remodeling activity alters chromatin structure and induces changes in transcription, DNA replication, DNA repair, or other processes that require remodeling. These events contribute to E4orf4 functions during virus infection and lead to cell death when E4orf4 is expressed alone. This figure is adapted from [57] with permission of Oxford University Press.

ACF is an ATP-dependent chromatin remodeling factor consisting of the SNF2h ATPase and the Acf1 regulatory subunit [56]. Both subunits were found to co-immunoprecipitate with E4orf4, and the PP2A C subunit associated with ACF in the presence of WT E4orf4 but not in its absence or in the presence of a mutant E4orf4 protein that could not bind PP2A [57]. Moreover, when chromatin proteins were fractionated by extraction in increasing salt concentrations, the PP2A-B55 subunit was enriched in higher salt fractions in the presence of E4orf4 compared to its distribution in the absence of E4orf4 or in the presence of the mutant E4orf4 protein. These results suggest that E4orf4 alters the chromatin-binding properties of PP2A, possibly by recruiting it to ACF. It was also demonstrated that the ACF complex was involved in E4orf4-induced cell death. Reduced SNF2h expression or expression of a dominant-negative catalytically-inactive SNF2h mutant inhibited E4orf4-induced cell death whereas, surprisingly, knockdown of Acf1 increased cell death. Knockdown of an Acf1 homolog, WSTF, which also associates with SNF2h, inhibited E4orf4-induced toxicity. The hypothesis proposed to explain these results inferred that the E4orf4-PP2A complex inhibited ACF and facilitated enhanced chromatin remodeling activities of other SNF2h-containing complexes, such as WSTF-SNF2h. An E4orf4-induced switch in chromatin remodeling may determine life *versus* death decisions [57] (Figure 3). This hypothesis predicts phosphorylation changes in ACF or ACF-associating proteins that could be important to E4orf4 function, but such alterations have not been reported yet. It is not likely, however, that E4orf4 recruits PP2A to a new substrate like ACF, with the sole purpose of inhibiting PP2A activity towards it.

### 3.3. The APC/C Ubiquitin Ligase Complex

A second potential E4orf4-PP2A target is the mitotic regulator APC/C. As described above (Section 2.5), work in yeast revealed that E4orf4 recruited PP2A to the APC/C complex and affected degradation of APC/C substrates [45,50]. As a result of the physical and functional interactions between E4orf4 and the APC/C, E4orf4 caused perturbations in the cell cycle which progressed to cell death. Genetic experiments revealed a functional interaction between the yeast B55 subunit, Cdc55, and the APC/C activating subunits Cdc20 and Cdh1 even in the absence of E4orf4 [45,75,76]. It was also shown that a PP2A complex containing the Cdc55 subunit dephosphorylated APC/C subunits [77]. It is possible therefore that E4orf4 may stabilize an existing transient interaction between PP2A and the APC/C [45]. It was reported that E4orf4 increased nuclear accumulation of Cdc55 [50], thus possibly enhancing the targeting of nuclear substrates, such as the APC/C by PP2A holoenzymes containing the B55/Cdc55 subunit. However, phosphorylation sites in the APC/C complex or its activating subunits, which may be affected by the E4orf4-PP2A complex, have not been identified to date. Interestingly, APC/C was found to be a target of many viral proteins and some of these proteins cause cytotoxicity specifically in tumor cells, providing evidence that targeting the APC/C could be exploited to selectively eliminate cancer cells [78].

### 3.4. The Golgi UDPase, Ynd1

Inducible expression of E4orf4 in the yeast *S. cerevisiae* was shown to result in PP2A-dependent irreversible growth arrest [18,45], suggesting that at least part of the E4orf4 signaling network responsible for induction of cell death is conserved from yeast to mammals. A further confirmation of the high degree of evolutionary conservation of E4orf4-induced cell death came from a genetic screen in yeast for E4orf4 mutants that lost their toxicity. These mutants were shown to have impaired abilities to bind PP2A and to induce cell death in mammalian cells [79]. Thus the results suggest that E4orf4-induced toxicity in yeast reflects accurately E4orf4-induced cell death in mammalian cells and therefore yeast may serve as a powerful genetic model system for the study of E4orf4-induced cell killing. Studies in this model organism revealed that not all of E4orf4-induced toxicity was generated via a B55/Cdc55-dependent pathway. Although much of the E4orf4 toxicity was abrogated in yeast cells lacking Cdc55, some residual growth inhibitory effect of E4orf4 could be detected [18,76]. Moreover, E4orf4 mutants that were deficient in PP2A binding could elicit low levels of toxicity [18]. A yeast screen for mediators of E4orf4 toxicity indeed demonstrated that a second E4orf4 partner, the Golgi apyrase Ynd1, contributed to E4orf4-induced irreversible growth arrest, and the contributions of Cdc55 and Ynd1 to this process were additive, indicating that they were not part of the same pathway [76].

Apyrases are nucleoside triphosphate diphosphohydrolases that hydrolyze a variety of nucleoside 5’-triphosphates and 5’ diphosphates with different nucleotide preferences. Most members of this family are integral membrane glycoproteins, and their catalytic sites face the extracellular medium or the lumen of intracellular organelles [80]. Ynd1 is a Golgi apyrase whose enzymatic activity is required for regulation of nucleotide-sugar import into the Golgi lumen. This protein is inserted in the Golgi membrane, its 500 N-terminal amino acids, including its catalytic domain, are located in the Golgi lumen, whereas its 113 C-terminal residues are found at the cytoplasmic face of the Golgi membrane [81,82].

Although Cdc55 and Ynd1 had an additive effect on E4orf4 toxicity, there was also a functional interaction between them, since deletion of *YND1* sensitized the cells to killing by E4orf4 in the presence of over-expressed *CDC55*. It was further shown that the Ynd1 and Cdc55 proteins interacted physically [76]. The physical and functional interactions between Ynd1 and Cdc55 together with their additive effects on E4orf4 toxicity are consistent with the hypothesis that the Ynd1-mediated E4orf4 function has PP2A-dependent as well as PP2A-independent aspects. Investigation of the role of mammalian Ynd1, Golgi UDPase, in E4orf4-induced cell death supported this possibility. On the one hand, E4orf4 was shown to stabilize the UDPase protein in a PP2A-dependent manner and the rise in UDPase levels increased E4orf4-induced cell death, whereas reduced UDPase expression inhibited E4orf4 toxicity. On the other hand, E4orf4 disrupted large molecular weight protein complexes containing the Golgi UDPase in a PP2A-independent manner [83]. A functional interaction between *YND1* and the APC/C activators, *CDC20* and *CDH1* was also observed, although the nature of this link is not currently understood [76].

Surprisingly, the Ynd1 cytosolic tail was found to mediate E4orf4-induced toxicity whereas the apyrase activity was dispensable for this process [76,84]. It was suggested that the Ynd1 cytosolic tail could potentially mediate E4orf4 toxicity by acting as a scaffold for a multi-protein complex which is targeted and disrupted by E4orf4, releasing some of its protein components [84]. The *Saccharomyces* Genome Database cites at least 10 membrane proteins, which physically associate with Ynd1, probably through its cytosolic tail. Six of these proteins associate with each other and may all be part of a large protein complex. Some of them participate in both early and late secretory pathways in yeast, and it is conceivable that E4orf4 targets a secretory protein complex anchored to the Ynd1 cytosolic tail. It was shown that E4orf4 affected protein trafficking in mammalian cells through its association with Src kinases ([85], Section 3.5). It is possible that in yeast cells, which lack Src family members, E4orf4 may utilize a backup mechanism which allows it to interact directly with components of the secretory pathway and to influence protein trafficking, thus further transducing its toxic signal [84]. This mechanism is likely used by E4orf4 in mammalian cells as well.

### 3.5. Src Family Kinases (SFKs)

When expressed alone, E4orf4 was found to associate with SFKs [14,66] but this interaction was not reported during virus infection. Absence or low levels of E4orf4-Src interaction during virus infection may stem from the inaccessibility of E4orf4 to Src, as E4orf4 is mostly nuclear in virus-infected cells [26]. E4orf4 interacts with less than 1-5% of endogenous Src proteins, depending on the cell type. This interaction is mediated by the kinase domain of Src and the E4orf4 ARM domain [14]. The Src‑binding domain of E4orf4 slightly overlaps with the PP2A binding domain, but E4orf4 complexes containing both PP2A and Src were detected, demonstrating that binding of these proteins to E4orf4 was not mutually exclusive [14]. Src binding is required for the cytoplasmic cell death program induced by E4orf4 [14]. The interaction of E4orf4 with SFKs leads to alterations in both protein partners. E4orf4 is phosphorylated on tyrosine residues within motifs that are typical of SFK substrates, including Tyr-26, -42, and -59 and these phosphorylation events affect E4orf4 cellular localization and cell death signaling [68]. Conversely, WT E4orf4 protein, but not E4orf4 mutants that are unable to bind SFKs, modulates SFK signaling. Thus, E4orf4 inhibits phosphorylation of substrates that are involved in SFK signaling to survival pathways, such as focal adhesion kinase (FAK) and ERK [14,66], and on the other hand it promotes SFK phosphorylation of substrates that are involved in regulation of actin dynamics, such as the F-actin-binding protein cortactin, JNK, and myosin light chain [14,66,86]. As a result, E4orf4 appears to recruit SFK signaling to stimulate actin-dependent membrane remodeling, leading to cell death through multiple mechanisms [87].

Following phosphorylation by SFKs, E4orf4 accumulates at cytoplasmic sites and induces the assembly of a juxtanuclear actin-myosin network. The juxtanuclear actin-myosin structures are connected to the cell cortex by actin cables, which converge on enlarged focal adhesions that reflect an increase in cell tension, [86,87]. Activation of the myosin II motor and the juxtanuclear increase in actin-myosin-based contraction drives E4orf4-induced blebbing, an early characteristic morphology that accompanies the E4orf4 cell death process [66].

The dramatic actin remodeling induced by E4orf4 requires two major pathways involving the family of Rho GTPases. The first is a Src-Rho-Rho kinase (ROCK) signaling pathway that activates JNK to phosphorylate Paxillin. Paxillin phosphorylation leads to deregulation of adhesion dynamics and disrupts tension homeostasis in the cell [88]. The second pathway consists of Cdc42, N-Wasp, and the Arp2/3 complex, and promotes actin polymerization on internal membranes and alterations in recycling endosome (RE) dynamics [86]. REs are a heterogeneous population of tubulovesicular endosomes usually concentrated in the pericentriolar region, which form the endocytic recycling compartment (ERC). RE trafficking is regulated by the small GTPase Rab11 and is involved in retrieval of internalized membranes and signaling molecules to the plasma membrane or to the trans-Golgi via retrograde membrane transport. Both pathways involving the Rho GTPases cooperate to transduce the cytoplasmic death-promoting activity of E4orf4, which acts via actin remodeling [86]. Indeed, inhibition of actin polymerization by low doses of drugs such as cytochalasin D reduced E4orf4-induced cell death, indicating a causal role for actin dynamics in this process [66,86].

A more detailed investigation of the role of actin remodeling and RE trafficking in E4orf4 death signaling [85] revealed that in the early stages of E4orf4-induced cell death, some SFKs, Cdc42, and actin interfered with the organization of the endocytic recycling compartment and enhanced RE transport to the Golgi apparatus while decreasing the recycling of protein cargos back to the plasma membrane. The resulting changes in Golgi membrane dynamics required actin-regulated Rab11a membrane trafficking and triggered scattering of Golgi membranes. This process functionally contributed to the progression of cell death as shown by the findings that Rab11a knockdown inhibited cell death as did knockdown of syntaxin-6, a TGN trafficking factor involved in fusion of RE with Golgi membranes, or overexpression of golgin-160, a Golgi matrix protein associated with Golgi dynamic changes during apoptosis. Thus, E4orf4 acts by recruiting SFK signaling to promote transport of REs to the Golgi where they may facilitate the dynamic rearrangement of membranes. It was suggested that E4orf4-induced assembly of the perinuclear actin network may be the outcome of polarized membrane traffic, which may facilitate delivery of actin-remodeling factors [85]. It is currently not known how fission of Golgi membranes brings about cell death, although an appealing suggestion was made whereby factors released from the Golgi may contribute to this process, similarly to factors released from the mitochondria [85].

In addition to its effects in the Golgi apparatus, E4orf4 was reported to cause dramatic changes in the morphology of mitochondria and to stimulate their mobilization to the polarized actin network using SFK- and Rab11a-dependent mechanisms. Based on these findings it was proposed that Rab11a may be responsible for regulation of both polarized membrane trafficking and mitochondrial dynamics during rearrangement of the cell to coordinate organelle functions with cytoskeletal dynamics [32]. It was recently realized that oxidative phosphorylation in the mitochondria plays a role in ATP production in cancer cells, as well as in proliferating cells and not just in quiescent cells [89]. The E4orf4-induced structural changes in mitochondria may lead to metabolic reprogramming which could contribute to the E4orf4-induced cell death process.

## 4. E4orf4-Induced Cell Death in an Animal Model

Because the mechanisms underlying PP2A-dependent E4orf4-induced toxicity appeared to be highly conserved in evolution from yeast to mammals, the effect of E4orf4 in a multicellular organism was investigated in another model organism, *Drosophila melanogaster*, which provides many genetic tools that facilitate the efficient investigation of regulatory pathways. The study of E4orf4 in *Drosophila* revealed that the characteristics of E4orf4-induced cell death in the fly were very similar to those in mammalian cells [22] (Figure 2). Thus in normal *Drosophila* tissues, E4orf4 induced low levels of cell killing, caused by both caspase-dependent and –independent mechanisms. *Drosophila* PP2A-B55 (*twins/abnormal anaphase resolution*) and Src64B contributed additively to this form of cell-death. However, this study, which addressed for the first time the consequences of E4orf4 expression in a whole multicellular organism, revealed a surprising finding: E4orf4 not only induced cell death but also inhibited classical apoptosis induced by the fly proapoptotic genes *reaper (rpr)*, *head involution defective (hid)*, and *grim*. E4orf4 also inhibited cell death induced by JNK signaling. However, whereas inhibition of *rpr*, *hid* and *grim* partially reduced cell killing, JNK inhibition did not diminish E4orf4-induced toxicity and even enhanced it. These results indicate that E4orf4-induced cell killing is a distinctive form of cell death that differs from classical cell death pathways induced by *rpr*/*hid*/*grim* or JNK signaling. Although E4orf4 appeared to inhibit JNK signaling in *Drosophila* [22], it was reported to activate JNK in certain transformed mammalian tissue culture cells [14,88]. This apparent contradiction can be explained by findings described previously, demonstrating that JNK signaling can be highly dependent on cellular context and on the nature of the stimulus [90,91,92]. Alterations of these conditions may impact the interaction between E4orf4 and the JNK pathway [22]. Future studies will have to determine whether JNK inhibition or activation by E4orf4 depend on the tumorigenic state of the cells, on the cell environment (monolayer or tissue) or on the type of organism studied.

The combination of both induction and inhibition of cell death by E4orf4 which was observed in normal *Drosophila* resulted in minor effects, leading to minimal tissue damage [22]. However, unpublished results from our laboratory indicated that expression of E4orf4 in cancer clones that were induced in larval eye discs by a combination of an activated oncogene and deletion of a tumor suppressor led to a dramatic reduction in cancer clone size and to significantly enhanced survival of adult flies that would not have emerged in the absence of E4orf4. These findings suggest one possible explanation for the differential effect of E4orf4 in normal and cancer cells. It can be hypothesized that E4orf4 is unable to inhibit apoptosis in cancer cells, thus inducing higher cell death levels [31]. This hypothesis is one of several possible explanations for enhanced cell killing by E4orf4 in cancer cells, which will have to be tested in the future (Section 2.1). As described above, E4orf4-mediated protection from toxicity was also observed in untransformed mammalian cells, albeit in the context of virus infection [4].

## 5. Perspectives

**Figure 4:**
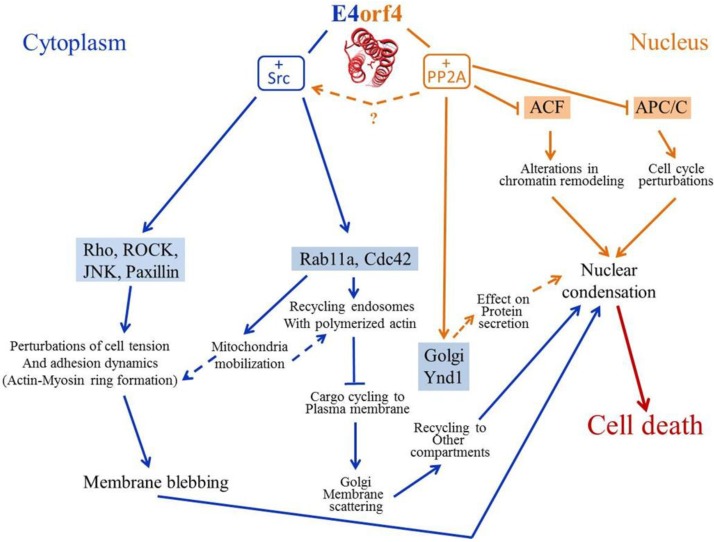
An integrated model of the mechanisms underlying E4orf4-induced cell death. E4orf4, in collaboration with its partners, PP2A and Src, induces alterations in the nucleus (chromatin remodeling, perturbations in cell cycle regulation) and in the cytoplasm (actin remodeling, changes in protein and membrane trafficking, changes in mitochondria morphology and mobilization), which result in blebbing, nuclear condensation and cell death: see the text for details. Arrows representing PP2A-dependent signaling are marked in orange and arrows showing Src-dependent signaling are marked in blue. Nuclear effectors are highlighted in orange and non-nuclear effectors are highlighted in blue. Connections in the E4orf4 network that were suggested but not proven yet are shown by discontinuous arrows. E4orf4 is represented by a structural model containing three alpha-helices as suggested by *ab initio* modeling [12]. This model was originally published in J. Biol. Chem. by Ben Horowitz, Rakefet Sharf, Meirav Avital-Shacham, Antonina Pechkovsky, and Tamar Kleinberger. 2013. 288: 13718-13727. © The American Society for Biochemistry and Molecular Biology.

E4orf4 research has progressed significantly in the past several years as summarized in Figure 4. Several mechanisms appear to contribute additively to E4orf4-induced cell death although their relative importance in this process may vary in different cells. However, many questions remain unanswered. Two major E4orf4 partners, PP2A and Src kinases were identified and appear to provide much of the contribution to E4orf4-induced cell death. However, several other E4orf4 partners, such as the Ynd1 Golgi UDPase, may make additional contributions. Downstream effectors of both PP2A and Src have been identified but a detailed analysis of the connectivity of the E4orf4 network is still lacking. Thus, for example, what are the direct targets of the E4orf4-PP2A complex in chromatin and in the APC/C and how exactly does their altered phosphorylation influence E4orf4-induced cell death? What is the molecular nature of the contribution of changes in protein trafficking and Golgi membrane disruption to nuclear condensation and cell death? Is there a crosstalk between PP2A and Src kinases during the E4orf4 cell death process? What is the contribution of Ynd1 and of other as yet undescribed E4orf4-associating proteins to induction of cell death? These are just a few examples of questions that remain open. Furthermore, our as yet unpublished results demonstrate that E4orf4 contributes to inhibition of the cell DNA damage response by Ad, and this function may also aid in cancer cell killing by E4orf4, requiring further study. Once the molecular signaling involved in E4orf4-induced cell death is further elucidated, it will be easier to compare the underlying mechanisms in normal and cancer cells and identify the reasons for the different susceptibility of these cells to E4orf4. Moreover, the E4orf4 cell death network should be investigated not only in tissue culture cells but also in animal models to provide additional insights. The knowledge obtained in such experiments will further aid in design of novel E4orf4-based cancer therapeutics.
